# Luteococcus struthionis sp. nov. and Lacrimispora struthionigena sp. nov. isolated from ostrich faeces

**DOI:** 10.1099/ijsem.0.007209

**Published:** 2026-06-22

**Authors:** Samuel L. Miller, R. Ty Young, Md. Mehedi Hasan, Julia M. Vinzelj, Noha H. Youssef, Mostafa S. Elshahed

**Affiliations:** 1 1 Department of Microbiology and Molecular Genetics, Oklahoma State University, Stillwater, OK, USA

**Keywords:** faeces, *Lacrimispora*, *Luteococcus*, ostrich, taxonomy, whole genome sequencing

## Abstract

Two mesophilic, Gram-stain-positive bacteria, OSA5^T^ and AGF001^T^, were isolated anoxically from ostrich faecal material. Phylogenetic analyses based on 16S rRNA gene and whole-genome sequences revealed that strains OSA5^T^ and AGF001^T^ formed distinct lineages within the genera *Luteococcus* and *Lacrimispora*, respectively. Strain OSA5^T^ showed the closest relatedness to *Luteococcus japonicus* DSM 10546^T^ and *Luteococcus sediminum* DSMZ 27277^T^ with 96.6% 16S rRNA gene sequence similarity. Strain AGF001^T^ was closely related to *Lacrimispora aerotolerans* DSM 5434^T^, with 97.4% 16S rRNA gene sequence similarity. Subsequent genomic analyses using overall genomic-based relatedness indices revealed that OSA5^T^ and AGF001^T^ shared average nucleotide identity and digital DNA–DNA hybridization values <95.0% and <70.0% with their close relatives, below the currently accepted thresholds for species-level delineation. The genomes of strains OSA5^T^ and AGF001^T^ were 3.2 Mbp and 5.4 Mbp, with G+C contents of 67.8 and 41.6 mol%, respectively. Based on phenotypic and genotypic data, strains OSA5^T^ and AGF001^T^ represent novel species in the genera *Luteococcus* and *Lacrimispora*, respectively; the names *Luteococcus struthionis* sp. nov. and *Lacrimispora struthionigena* sp. nov. are proposed. The type strains are OSA5^T^ (=CCM 9469^T^=CCUG 78312^T^=CECT 31229^T^=DSM 119679^T^) and AGF001^T^ (=CCM 9472^T^=CCUG 78314^T^=CECT 31227^T^=DSM 119664^T^), respectively.

## Data Availability

The GenBank/EMBL/DDBJ accession numbers for the 16S rRNA gene sequences of strains OSA5^T^ and AGF001^T^ are PV166456.1 and PV166457.1. The GenBank/EMBL/DDBJ accession numbers for the whole-genome sequences of strains OSA5^T^ and AGF001^T^ are GCA_048595755.1 and GCA_048627165.1. The type strain OSA5^T^ has been deposited in the Czech Collection of Microorganisms (accession number: CCM 9469^T^), the Culture Collection University of Gothenburg (accession number: CCUG 78312^T^), the Spanish Type Culture Collection (accession number: CECT 31229^T^) and the German Collection of Microorganisms and Cell Cultures GmbH (accession number: DSMZ 119679^T^). The type strain AGF001^T^ has been deposited in the Czech Collection of Microorganisms (accession number: CCM 9472^T^), the Culture Collection University of Gothenburg (accession number: CCUG 78314^T^), the Spanish Type Culture Collection (accession number: CECT 31227^T^) and the German Collection of Microorganisms and Cell Cultures GmbH (accession number: DSMZ 119664^T^)

## Introduction

The ostrich (*Struthio camelus*) is the largest extant herbivorous flightless member of the infraclass *Palaeognathae*, compared to the other members within *Palaeognathae*: rheas (*Rhea*), cassowaries (*Casuarius*), emus (*Dromaius*) and kiwis (*Dromaius*). Ostriches exhibit distinct physiological and anatomical adaptations. Their hindgut-fermenting digestive system [1], characterized by an enlarged cecum and colon [2], facilitates the breakdown of fibrous plant materials (primarily consumed grasses, shrubs and succulents [3,4]) through the enzymatic activities of lignocellulose-degrading micro-organisms [5,6]. This process is essential for extracting energy from otherwise indigestible plant material [7]. Resident microbial communities in the ostrich digestive system produce short-chain fatty acids such as acetate, propionate and butyrate, which serve as vital energy sources for the host [8,10]. Despite increasing interest in the gut microbiomes of avian species, research on the ostrich microbiota remains relatively limited. Studies employing culture-independent approaches have reported the predominance of specific bacterial taxa within the ostrich gut (mainly members of the phylum *Verrucomicrobiota* and class *Clostridia* in juveniles and adults [11,12]. Recently, a study on the anaerobic gut fungi (*Neocallimastigomycota*) communities in domestic ostriches revealed distinct anaerobic gut fungi populations compared to those previously studied in other herbivorous animals [13].

The genus *Luteococcus* (phylum *Actinomycetota,* class *Actinomycetes*, order *Propionibacteriales*, family *Propionibacteriaceae*) was formally described in 1994 [14] and currently comprises four validly published species (https://lpsn.dsmz.de/genus/*Luteococcus*, October 2025). Collectively, *Luteococcus* species are Gram-stain-positive, facultatively anaerobic, coccus-shaped, non-endospore-forming bacteria with a high DNA G+C content [14] (67.8–70.5 mol%, Table 1). These taxa have been isolated from terrestrial [14] and aquatic [15] environments, as well as from humans [16,17]. However, one metagenomic study generated a metagenome-assembled genome ‘*Candidatus Luteococcus avicola*’ from chickens’ faecal samples [18].

**Table 1.** General genomic features of OSA5^T^ (top panel), AGF001^T^ (bottom panel) and their close relatives

Characteristic	OSA5^T^	*Luteococcus japonicus*	*Luteococcus peritonei*	*Luteococcus sanguinis*
**Genome accession**	GCA_048595755.1	GCF_003752415.1	GCF_039526385.1	GCF_039527675.1
**DNA G+C (mol%**)	67.8	67.8	70.5	68.2
**Genome size (Mbps**)	3.2	3.5	3.3	2.9
**No. of contigs**	1	1	50	92
**No. of CDSs**	3,021	3,344	3,109	2,752
**Characteristic**	**AGF001^T^**	***Lacrimispora aerotolerans***	***Lacrimispora xylanolytica***	***Lacrimispora amygdalina***
**Genome accession**	GCA_048627165.1	GCF_000687555.1	GCF_002934545.1	GCF_003609635.1
**DNA G+C (mol%**)	41.5	42.4	41.9	40.2
**Genome size (Mbps**)	5.4	4.7	5.6	4.6
**No. of contigs**	3	50	33	35
**No. of CDSs**	5,045	4,397	5,340	4,311

Strains (top panel): OSA5T; *Luteococcus japonicus* DSM 10546T; *Luteococcus peritonei* CCUG 38120T; *Luteococcus sanguinis* CCUG 33897T; no whole-genome sequence was available for the type strain of *Luteococcus sediminum*.

Strains (bottom panel): AGF001T; *Lacrimispora aerotolerans* DSM 5434T; *Lacrimispora xylanolytica* ATCC 49623T; *Lacrimispora amygdalina* BR-10T.

CDSs, coding sequences.

The genus *Lacrimispora* (phylum *Bacillota*, class *Clostridia*, order *Eubacteriales*, family *Lachnospiraceae*) was given taxonomic standing in 2020 [19] and currently comprises 11 validly published species (https://lpsn.dsmz.de/genus/*Lacrimispora*, October 2025). Members of this genus are structurally Gram-stain-positive, obligately anaerobic, rod-shaped, spore-forming bacteria, with a low DNA G+C content [19] (40.2–42.4 mol%, Table 1). *Lacrimispora* have been primarily isolated from non-host associated habitats, e.g. fermented cabbage, sewage sludge, pickled mustard, woodchips, freshwater sediments, although association with hosts has also been reported, e.g. *Lacrimispora aerotolerans*, which was isolated from the rumen of a sheep [20]. To date, neither of these genera contains a validly published species from an avian host.

This study describes two novel *Luteococcus* and *Lacrimispora* species isolated from ostrich faecal material. This work contributes to the broader effort to elucidate avian gut microbial diversity; additionally, it expands our current understanding of the diversity of *Luteococcus* and *Lacrimispora*, as this represents the first instance of novel bacterial taxa descriptions for these genera from avian hosts. This study employs a polyphasic taxonomic approach to describe two novel species obtained from ostrich faeces: *Luteococcus* strain OSA5^T^ and *Lacrimispora* strain AGF001^T^. Strains OSA5^T^ and AGF001^T^ represent novel species within their respective genera, and the names *Luteococcus struthious* sp. nov. and *Lacrimispora struthionigena* sp. nov. are herein proposed.

## Isolation of bacterial strains and culture conditions

Strains OSA5^T^ and AGF001^T^ were isolated from ostrich faecal material collected from a private farm near Catoosa, Oklahoma (34.486589, −98.226482) on 1 June 2022. The faecal material was placed in an anaerobic chamber (Coy Laboratory Products, Inc., Grass Lake, MI) and serially diluted in anoxic PBS (1 x). Isolation procedures were conducted using either an initial anoxic enrichment approach followed by plating, or a direct plating approach from the original samples. Anoxic enrichments were prepared by serially diluting faecal material, which was inoculated into rumen fluid (RF) media containing 0.5% (w/v) cellobiose, as previously described [21], and sub-cultured upon visually observing signs of visible growth (24–48 h). Subsequently, an aliquot of RF media was serially diluted in anoxic PBS (1 x) and plated onto tryptic soy agar (TSA) plates supplemented with 5% (v/v) defibrinated sheep’s blood (TSAB). For the direct plating approach, (0.1 ml) serially diluted faecal material (1 : 10) was spread onto Columbia Agar supplemented with 5% (v/v) defibrinated sheep’s blood (CAB), Reasoner’s 2A Agar (R2A), TSA and MacConkey agar. All plates were incubated under strictly anaerobic conditions for 2 weeks in an anaerobic chamber (Coy Laboratory) filled with 90% N_2_ and 10% H_2_ at 37 °C. Individual colonies with differing morphological profiles were further sub-cultured until pure isolates were obtained, as evidenced by phase-contrast microscopy (BX51, Olympus, Centre Valley, PA).

The Gram stain reaction was determined using the Gram Stain Kit (Becton Dickinson,

Franklin Lakes, NJ), following the manufacturer’s instructions. Phase-contrast microscopy (BX51, Olympus, Centre Valley, PA) was used to assess cell shape, motility and endospore production. Cell size for OSA5^T^ and AGF001^T^ was determined by scanning electron microscopy with a FEI Quanta 600 field-emission gun environmental scanning electron microscope (Thermo Fisher, Waltham, MA).

Strain OSA5^T^ was isolated on a CAB by direct plating, whereas AGF001^T^ was isolated on TSAB following an enrichment procedure. After primary isolation, OSA5^T^ and AGF001^T^ were maintained under anoxic conditions in anoxic tryptic soy broth (TSB) or TSAB at 37 °C. Isolates OSA5^T^ and AGF001^T^ were preserved in Microbank cryogenic beads (Prolab Diagnostics, Georgetown, TX) according to the manufacturer’s instructions and stored at −80 °C. The cells of OSA5^T^ are Gram-stain-positive cocci, measuring 0.8–0.9 µm in diameter and occurring in pairs, tetrads or clusters (Fig. S1a, b, available in the online Supplementary Material). Colonies of OSA5^T^ on CAB medium were opaque, off-white, mucoid and circular on the agar surface after 48 h at 39 °C. The cells of AGF001^T^ are Gram-stain-negative rods, measuring 3.5 µm in height × 0.5 µm in length (Fig. S1c, d). Colonies on TSAB medium were white, circular and convex after 48 h at 37 °C.

## 16S rRNA gene-based phylogenetic analysis

Near full-length 16S rRNA gene sequences were obtained for OSA5^T^ and AGF001^T^ using Sanger sequencing [22] at the Oklahoma State University Biochemistry and Molecular Biology core sequencing facility. The 16S rRNA gene sequences were queried using a blast search against the sequences available on the EzBioCloud server (https://www.ezbiocloud.net/) [23,25]. The 16S rRNA gene sequences of OSA5^T^ and AGF001^T^, as well as those of closely related species, were downloaded from the EzBioCloud server [24]. These sequences were then aligned using muscle [26], and phylogenetic trees were reconstructed using the maximum-likelihood method [27] and the Tamura-Nei substitution model [28]with 1,000 bootstrap replications. The trees were visualized using mega 12 [29].

**Fig. 1.**
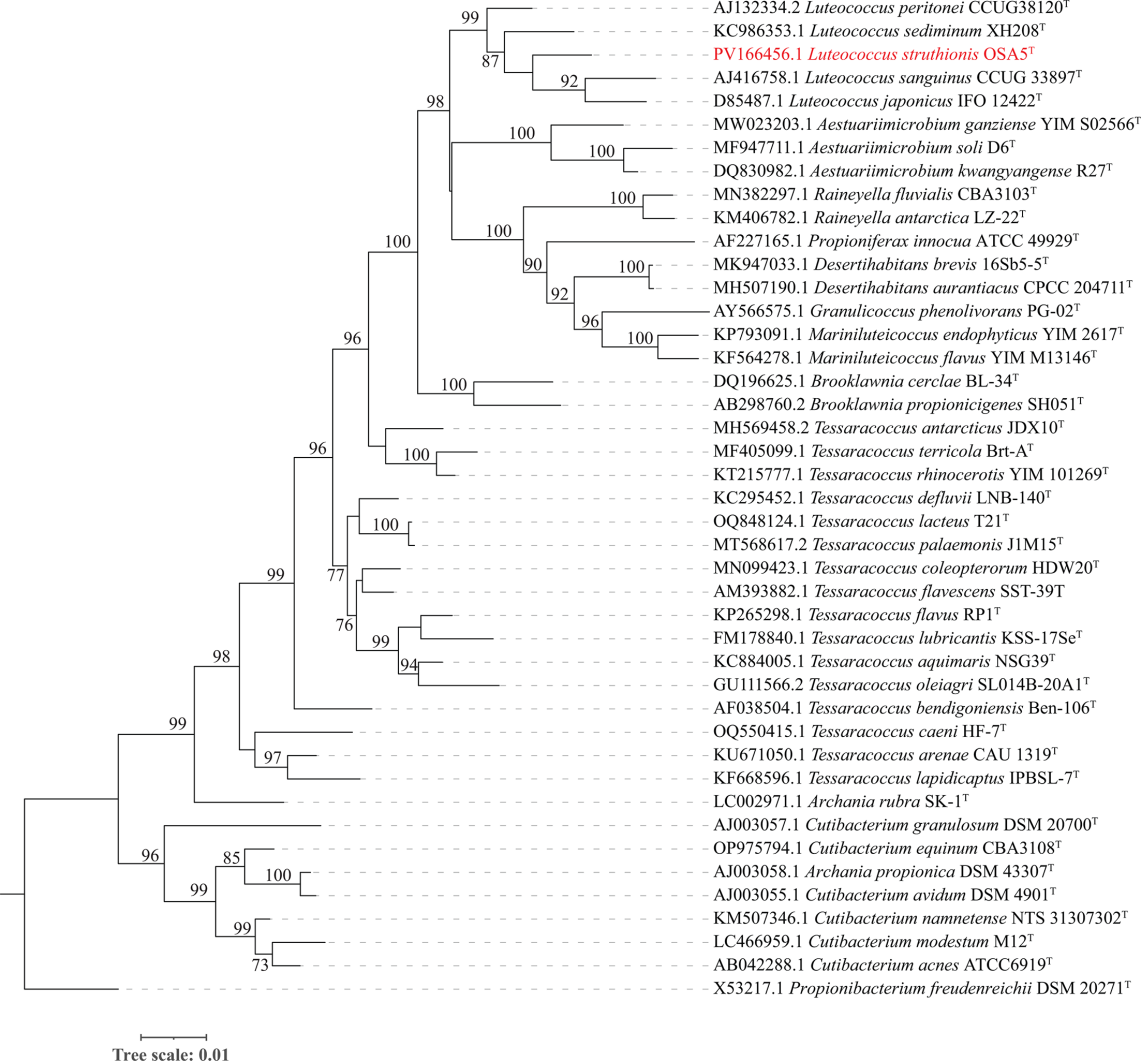
Phylogenetic tree based on 16S rRNA gene sequences showing the relationship between OSA5^T^ and other members of the family *Propionibacteriaceae*. Bar, 0.02 substitutions per nucleotide position.

**Fig. 2.**
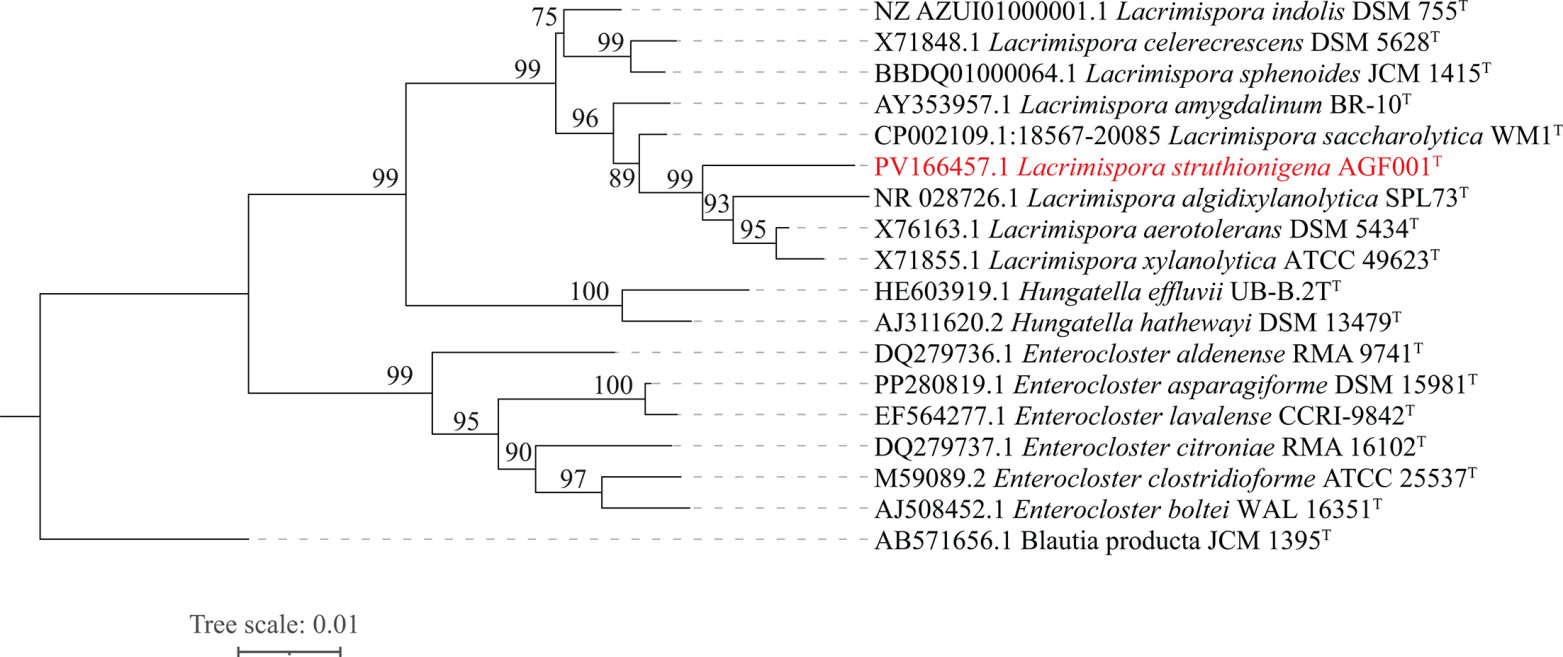
Phylogenetic tree based on 16S rRNA gene sequences showing the relationship between AGF001^T^ and other members of the family *Lachnospiraceae*. Bar, 0.02 substitutions per nucleotide position.

The results of 16S rRNA gene phylogenetic analyses indicated that OSA5^T^ was most closely related to *Luteococcus japonicus* and *Luteococcus sediminum* (96.6%), whereas AGF001^T^ was most closely related to *Lacrimispora aerotolerans* (97.4%) and *Lacrimispora xylanolytica* (96.8%) (Table 2). These values are below the currently accepted threshold of 98.7%, which is now routinely used to demarcate this taxonomic rank [30]. Additionally, the topologies of the associated phylogenetic trees constructed using the maximum-likelihood method [27] support the proposals that OSA5^T^ (Fig. 1) and AGF001^T^ (Fig. 2) represent novel species within *Luteococcus* and *Lacrimispora*, respectively.

**Table 2.** Pairwise comparison of 16S rRNA gene similarity and overall genomic relatedness indices between OSA5^T^ (top panel), AGF001^T^ (bottom panel) and their close relatives Strains: (top panel) OSA5^T^; *Luteococcus japonicus* DSM 10546^T^; *Luteococcus sediminum* XH208^T^; *Luteococcus peritonei* CCUG 38120^T^; *Luteococcus sanguinis* CCUG 33897^T^; (bottom panel) AGF001^T^; *Lacrimispora aerotolerans* DSM 5434^T^; *Lacrimispora xylanolytica* ATCC 49623^T^; *Lacrimispora amygdalina* BR-10^T^

Strain	OSA5^T^	*Luteococcus japonicus*	*Luteococcus sediminum*	*Luteococcus peritonei*	*Luteococcus sanguinis*
**16S accession**	PV166456	D85487	KC986353	AJ132334	AJ416758
**16S similarity (%**)	100.0	96.6	96.6	96.4	96
**Genome accession**	GCA_048595755.1	GCF_003752415.1	NR	GCF_039526385.1	GCF_039527675.1
**OrthoANIu (%**)	100.0	80.2	NR	78.5	75.4
**dDDH (%**)	100.0	23.8	NR	22	21
–	**AGF001^T^**	***Lacrimispora aerotolerans***	***Lacrimispora xylanolytica***	***Lacrimispora amygdalina***	–
**16S accession**	PV166457	X76163	X71855	AY353957	–
**16S similarity (%**)	100.0	97.4	96.8	96.6	–
**Genome accession**	GCA_048627165.1	GCF_000687555.1	GCF_002934545.1	GCF_003609635.1	–
**OrthoANIu (%**)	100.0	75.3	75.4	87.1	–
**dDDH (%**)	100.0	19.8	19.6	32.5	–

NR, not reported; no whole-genome sequence was available for *Luteococcus sediminum* XH208T .

## Whole-genome sequencing, phylogenomic analysis and *in silico* metabolic predictions

Cell biomass was harvested from an overnight culture and stored in DNA/RNA Shield (Zymo, Irvine, CA). Subsequent DNA extraction, sequencing and custom analysis and annotation were conducted using the services of a commercial provider (Plasmidsaurus, Eugene, OR) as previously described [31]. Completeness and contamination assessment of the obtained genomic assemblies were conducted using CheckM v1.2.2 [32]. Genome annotation was performed using the NCBI Prokaryotic Genome Annotation Pipeline v 6.7 [33]. Additional whole-genome-based phylogenetic analysis of OSA5^T^ and AGF001^T^ was performed with the Genome Taxonomy Database Toolkit (GTDB-Tk) [34], employing a concatenated alignment of 120 single-copy genes [34]. A phylogenomic tree was constructed using the maximum-likelihood approach [27] with RAxML, employing the PROTGAMMABLOSUM62 model and default parameters [35]. Trees were visualized using the Interactive Tree of Life v6 [36]. The taxonomic status of OSA5^T^ and AGF001^T^ was further confirmed by comparing overall genomic relatedness indices (OGRIs) according to the most recent proposed minimal standards for the use of genome data for the taxonomy of prokaryotes [37]. Specifically, average nucleotide identity (ANI) was calculated using EzBioCloud’s ANI Calculator, which uses the OrthoANIu algorithm [24,38]. The Genome-to-Genome Distance Calculator [39,40] v3.0 was used to determine digital DNA–DNA hybridization (dDDH). Carbohydrate-active enzyme (CAZyme) analysis was performed using the dbCAN3 meta-server [41,42] run through the Kbase [43] web server.

Strains OSA5^T^ and AGF001^T^ genome sizes were 3.2 and 5.4 Mbp, respectively. The DNA G+C content was determined to be 67.8 and 41.6 mol%, respectively. Genome quality metrics obtained from the NCBI PGAP CheckM analysis (v1.2.2) show high completeness (97.8%) and low contamination (3.2%) for OSA5^T^, as well as for AGF001^T^, with completeness and contamination levels of 98.7 and 3.7%, respectively. As such, both genomes were deemed acceptable for inclusion in taxonomic and phylogenomic studies [32]. Of the 3,021 total predicted genes for OSA5^T^, 2,926 were protein-coding sequences, 9 rRNA genes and 48 tRNAs. For AGF001^T^, a total of 5,045 genes were predicted, of which 4,913 were protein-coding sequences, 18 rRNA genes and 69 tRNAs were identified. The general genotypic characteristics of OSA5^T^ and AGF001^T^ and their close relatives are given in Table 2.

**Fig. 3.**
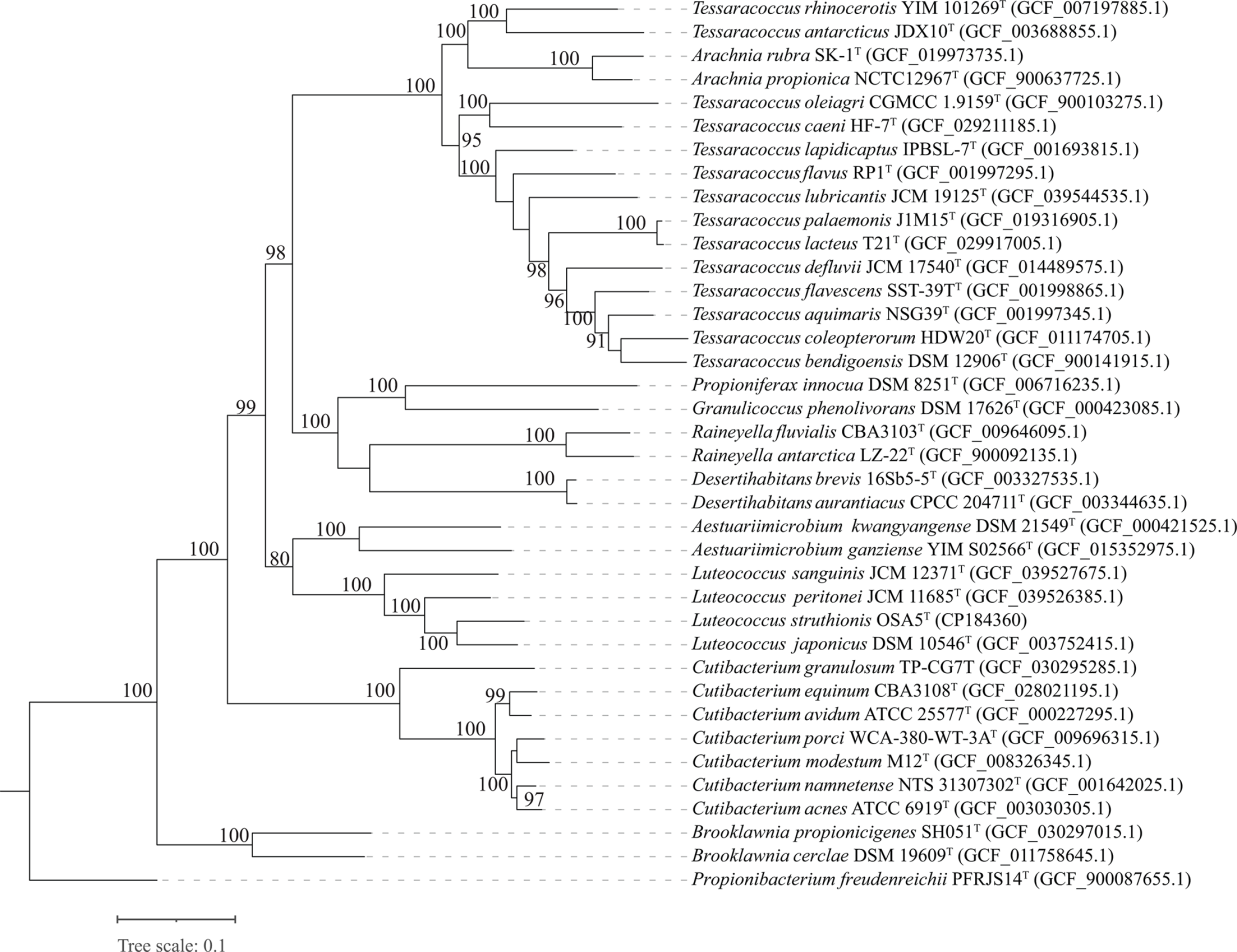
Core-genome phylogenomic tree using a concatenated alignment of 120 single-copy gene sequences showing the relationship between OSA5^T^ and other members of the family *Propionibacteriaceae*. Bar, 0.1 substitutions per site.

**Fig. 4.**
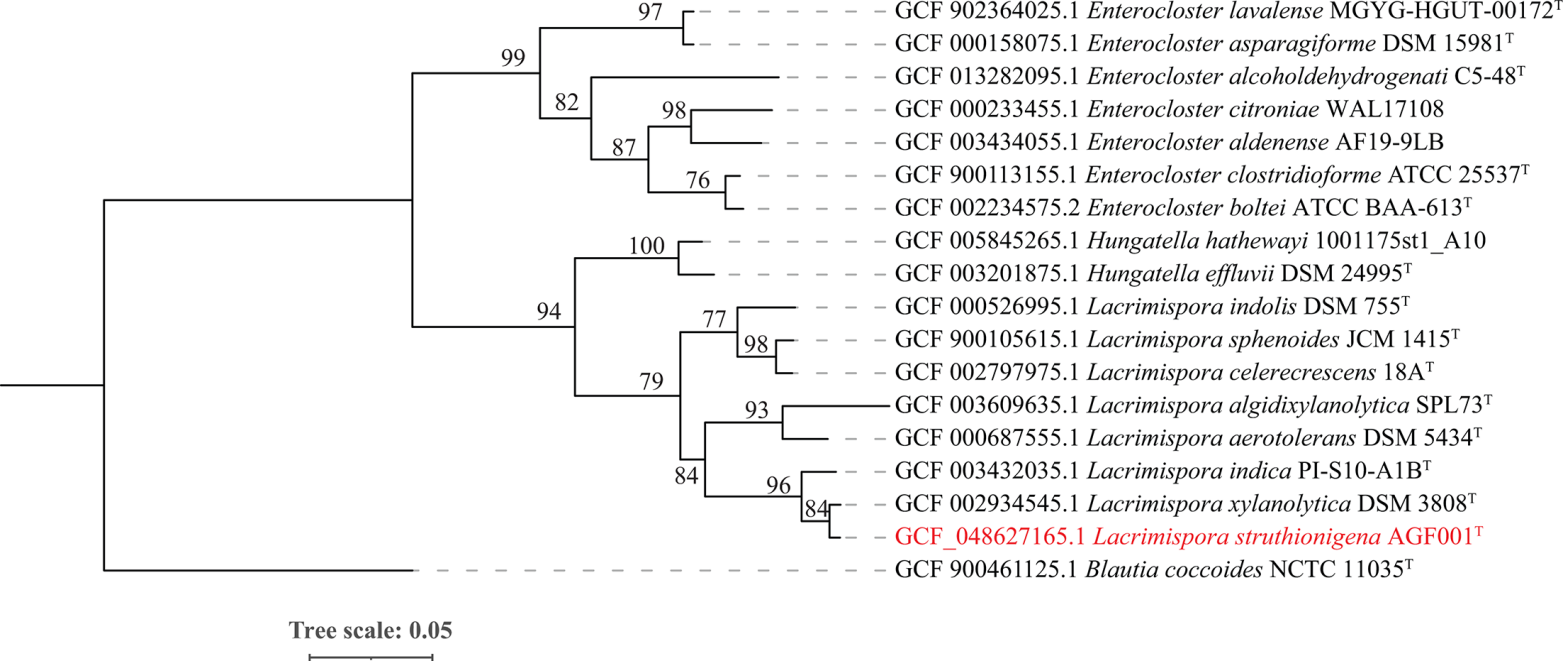
Core-genome phylogenomic tree using a concatenated alignment of 120 single-copy gene sequences showing the relationship between AGF001^T^ and other members of the *Lachnospiraceae*. Bar, 0.02 substitutions per site.

Phylogenomic analysis confirmed the affiliation of strain OSA5^T^ with the genus *Luteococcus*, clustering most closely with *Luteococcus japonicus* (GCF_003752415.1) (Fig. 3), as well as the affiliation of strain AGF001^T^ with the genus *Lacrimispora*, clustering most closely with *Lacrimispora xylanolytica* (GCF_002934545.1) (Fig. 4). Such topology was in concordance with the analyses of 16S rRNA gene-based phylogenies (Figs 1 and 2). OGRIs for species delineation revealed ANI values of <95.0% and dDDH values of <70.0% for OSA5^T^ and AGF001^T^ and their closest relatives (Table 1). These values are lower than the proposed thresholds for species-level delineation (95.0–96.0% and 70.0%, respectively) [30,39, 44]. Collectively, these results strongly indicate that both strains represent two novel, distinct species within the genera *Luteococcus* and *Lacrimispora*.

*In silico* prediction of metabolic capacities and physiological preferences of the OSA5T genome revealed characteristics typical of members of the genus *Luteococcus* (Table S1). This included a facultative anaerobic heterotrophic lifestyle, including pathways indicative of aerobic growth (Embden-Meyerhof-Parnas pathway, pyruvate-ferredoxin/flavodoxin oxidoreductase for pyruvate oxidative decarboxylation, a complete TCA cycle with a-ketoglutarate dehydrogenase and a complete electron transport system with NADH-quinone oxidoreductase [EC:7.1.1.2], succinate dehydrogenase [EC:1.3.5.1], cytochrome bc1 complex [EC:7.1.1.8] and cytochrome c oxidases [EC:7.1.1.9], in addition to the F-type ATPase for oxidative phosphorylation), as well as capacities for fermentation and substrate-level phosphorylation using the phosphate acetyltransferase-acetate kinase pathway, acetyl-CoA synthetase [EC:6.2.1.1], succinyl-CoA:acetate CoA-transferase [EC:2.8.3.18] and acetyl-CoA synthetase [EC:6.2.1.1] for acetyl-CoA conversion to acetate, pyruvate fermentation to formate via formate C-acetyltransferase [EC:2.3.1.54], pyruvate fermentation to l-lactate via l-lactate dehydrogenase [EC:1.1.1.27] and ethanol fermentation. On the other hand, analysis of the AGF001T genome revealed characteristics typical of members of the genus *Lacrimispora* (Table S1). This included obligate anaerobic growth (evidenced by absence of pyruvate dehydrogenase and alpha-ketoglutarate dehydrogenase, the oxidative branch of the PPP, as well as the complete absence of complexes I-IV of the electron transport chain). Energy production is expected to happen via fermentation and substrate-level phosphorylation as the genome encodes phosphate acetyltransferase-acetate kinase pathway and means for pyruvate fermentation to ethanol and l-lactate.

Analysis of the CAZyme repertoire in both genomes identified 248 glycoside hydrolases (GHs), 6 carbohydrate esterases and 1 polysaccharide lyase family in strain OSA5^T^, and 579 GHs, 23 carbohydrate esterases and 34 polysaccharide lyase families in strain AGF001^T^ (Fig. S2). The majority of the CAZyome in OSA5^T^ (77.9%) was comprised of amylases belonging to the GH13 family. When compared with two other *Luteococcus* species with sequenced genomes available in NCBI RefSeq [*Luteococcus japonicus* (GCF_003752415.1) and *Luteococcus japonicus* LSP Lj1 (GCF_900163845.1)], the same pattern was observed, with 78.3 and 80.8%of the total CAZyome belonging to GH13 family, respectively (Fig. S2). On the other hand, the majority of the CAZyome in AGF001^T^ consisted of amylases in the GH13 family (34.0% of total GHs) and cellulose- and hemicellulose-degrading enzymes in the GH43 family GH1 and GH5 families (42.5%). When compared with two other *Lacrimispora* species with sequenced genomes available in NCBI RefSeq [*Lacrimispora amygdalina* (GCF_900205965.1), *Lacrimispora celerecrescens* (GCF_900185635.1), *Lacrimispora saccharolytica* (GCF_016906045.1) and *Lacrimispora sphenoides* (GCF_900105215.1)], a similar pattern was observed (Fig. S2).

## Morphological and physiological characteristics

Aerotolerance was determined by placing cultures on CAB or TSAB under aerobic conditions for 1 week at 37 °C. A catalase test was performed as previously described [45]. Temperature ranges for growth were determined in TSB at 4 °C, 20 °C, 25 °C, 37 °C, 45 °C, 50 °C and 55 °C. Salt tolerance was determined in TSB at 0.5% (w/v) and between 1.0% (w/v) and 10.0% (w/v), in increments of 1.0% (w/v); pH ranges for growth were determined between 3.0 and 10.0, in increments of 1.0, and all tests were performed in duplicate. For the pH ranges, the Good’s buffers were prepared as previously described [46]. Optimal growth conditions were determined by optical density at 600 nm using a Multiskan Go Microplate Spectrophotometer (Thermo Fisher Scientific, Waltham, MA) or a Spectronic 20D+ (Milton Roy, Ivyland, PA). An increase in optical density of 600 nm greater than 0.1 after 5 days of incubation was considered growth.

Additional physiological and biochemical characteristics, such as carbon substrate utilization and chemical susceptibility, were determined using the GENIII microplate system (BIOLOG, Hayward, CA), the Anaerobe Identification Test Panel AN MicroPlate (Biolog, Hayward, CA), ID 32 Staph (bioMérieux, Durham, NC), API CORYNE (bioMérieux, Durham, NC), API Rapid ID32 A (bioMérieux, Durham, NC) and the API ZYM kit (bioMérieux, Durham, NC), according to the manufacturers’ protocols.

OAS5^T^ cells were negative for motility and endospore production. OSA5^T^ was facultatively anaerobic, growing when the media were exposed to air. Cells were positive for catalase and amylase. OSA5^T^ grew between 4 °C and 45 °C, with an optimum growth at 39 °C. OSA5^T^ could grow at NaCl concentrations between 0.5 and 9.0 % (w/v), with optimal growth occurring at 3.0 % (w/v) NaCl. OSA5^T^ grew at pH values between 5.0 and 7.0, with an optimum pH of 6.0.

Strain OSA5^T^ utilized Glucose, Fructose, Ribose, Xylose, Maltose, Sucrose, Melibiose, Gentiobiose, Stachyose, Pectin, Inosine, Mannitol, Sorbitol, Arabitol, Myo-inositol, Glycerol, Esculin, Gelatin, β-methyl-d-glucoside, d-salicin, *N*-acetyl neuraminic acid, Glucuronamide, Acetic acid, Propionic acid, Sodium butyrate, Mucic acid, Bromo-succinic acid, α-hydroxybutyric acid, α-ketobutyric acid, l-lactic acid, l-malic acid and l-alanine for growth. No growth occurred with Galactose, Mannose, Rhamnose, Fucose, Lactose, Cellobiose, Trehalose, Turanose, Raffinose, Glycogen, Dextrin, 3-methyl glucose, *N*-acetyl-d-glucosamine, *N*-acetyl-d-galactosamine, *N*-acetyl-β-d-mannosamine, Glucose-6-phosphate, Fructose-6-phosphate, l-butyric acid, Formic acid, Citric acid, Quinic acid, Galacturonic acid, d-gluconic acid, d-saccharic acid, l-galactonic acid lactone, β-hydroxy-d, α-ketoglutaric acid, Acetoacetic acid, d-lactic acid methyl ester, d-malic acid, l-arginine, l-aspartic acid, d-aspartic acid, l-glutamic acid, l-histidine, l-pyroglutamic acid, γ-aminobutyric acid or Glycyl-l-proline (Table S2).

In the chemical sensitivity assays, strain OSA5^T^ grew in the presence of 1.0% sodium lactate, lithium chloride, potassium tellurite, sodium bromate, Tween 40, nalidixic acid and aztreonam. No growth occurred in the presence of fusidic acid, troleandomycin, rifamycin SV, minocycline, lincomycin, guanidine HCl, niaproof 4, vancomycin, tetrazolium violet or tetrazolium blue.

Enzymatic assays for OSA5^T^ detected positive reactions for Alkaline phosphatase, Esterase (C4), Esterase lipase (C8), Leucine arylamidase, Trypsin, α-Chymotrypsin, Acid phosphatase, β-Glucuronidase, β-Galactosidase, α-Glucosidase, Arginine arylamidase, Pyrazinamidase, Pyrrolidonyl arylamidase, β-*N*-acetylglucosaminidase and Catalase. In contrast, negative results were obtained for lipase (C14), valine arylamidase, cystine arylamidase, naphthol-AS-BI-phosphohydrolase, α-Galactosidase, β-glucosidase, *N*-acetyl-β-glucosaminidase, α-mannosidase, α-fucosidase and Pyrrolidonyl arylamidase.

Distinguishing phenotypic characteristics between strains OSA5^T^ and their close relatives are listed in Table 3. Compared to other members of the genus *Luteococcus*, strain OSA5^T^ exhibited some unique substrate utilization patterns as well as enzymatic activities profiles. Specifically, strain OSA5^T^ was unique in its ability to utilize ribose and xylose for growth, as well as its lack of α-Galactosidase, β-Galactosidase and β-glucosidase activities.

**Table 3.** Morphological and physiological differential characteristics of OSA5^T^ (top panel), AGF001^T^ (bottom panel) and their close relatives

Characteristic	OSA5^T^		*Luteococcus japonicus*		*Luteococcus sediminum*		*Luteococcus peritonei*		*Luteococcus sanguinis*
**Cell shape**	Cocci		Cocci		Cocci		Pleomorphic rod		Cocci
**Cell size (µm**)	0.8–0.9		0.7–1.0		0.2–0.4		NR		NR
**Temperature range (°C**)	20–50		12–38		04–37		NR		NR
**Temperature optimum (°C**)	39		26–28		28		NR		NR
**Salinity range % (w/v**)	0.5–9.0		0.0–5.0		0.0–10.0		NR		NR
**Salinity optimum % (w/v**)	3.0		NR		2.0–3.0		NR		NR
**pH range**	5.0–7.0		NR		6.0–8.0		NR		NR
**pH optimum**	6.0		NR		7.0		NR		NR
**α-Galactosidase**	−		+		+		+		+
**β-Glucosidase**	−		+		+		+		+
**Ribose**	+		−		−		−		−
**Xylose**	+		−		−		−		−
**Isolation source**	Ostrich faeces		Soil		Subseafloor sediment		Human peritoneum		Human blood
**Characteristic**		**AGF001^T^**		***Lacrimispora aerotolerans***		***Lacrimispora xylanolytica***		***Lacrimispora amygdalina***	
**Cell width (µm**)		0.5		0.6		0.8		0.6	
**Cell shape**		Rod		Rod		Rod		Rod	
**Motility**		+		+		+		−	
**Temperature range (°C**)		20–45		NR		NR		17–43	
**Temperature optimum (°C**)		37		38		35		37	
**Salinity range % (w/v**)		0.5–2.0		NR		NR		NR	
**Salinity optimum % (w/v**)		0.5		NR		NR		NR	
**pH range**		6.0–8.0		NR		NR		6.0–8.8	
**pH optimum**		7.0		NR		7.2		7.4	
**Fructose**		–		+		+		+	
**Mannose**		–		+		+		+	
**Salicin**		–		+		+		+	
**Raffinose**		–		+		+		+	
**Isolation source**		Ostrich Faeces		Sheep rumina		Decayed wood chips		sewage sludge	

Strains (top panel): OSA5T (data from this study); *Luteococcus japonicus* DSM 10546T*; *Luteococcus sediminum* XH208T [15]; *Luteococcus peritonei* CCUG 38120T*; *Luteococcus sanguinis* CCUG 33897T*; NR, not reported. The following characteristics were shared among all isolates for which data were reported: Gram-stain-reaction (+), endospore production (-) and motility (-). *The α-Galactosidase and β-glucosidase data for *Luteococcus japonicus* DSM 10546T, *Luteococcus peritonei* CCUG 38120T and *Luteococcus sanguinis* CCUG 33897T were obtained from https://bacdive.dsmz.de/.

Strains (bottom panel): AGF001T (data from this study); *Lacrimispora aerotolerans* DSM 5434T [20]; *Lacrimispora xylanolytica* ATCC 49623T[47]; 4*, Lacrimispora saccharolytica* WM1T [48]; NR, not reported. The following characteristics were shared among all isolates for which data were reported: Gram-stain reaction (-) and endospore production (+).

Strain AGF001^T^ was obligately anaerobic, failing to grow when the media was exposed to air. Cells were positive for motility and endospore production, with a subterminal visible in phase contrast microscopy. Strain AGF001^T^ grew between 20 °C and 45 °C with an optimum growth at 37 °C. Strain AGF001^T^ could grow at NaCl concentrations between 0.5 and 2.0 % (w/v), with an optimal growth occurring at 0.5 % (w/v) NaCl. AGF001^T^ grew at pH values between 6.0 and 8.0 with an optimum pH of 7.0.

Strain AGF001^T^ utilized 2′-Deoxy Adenosine, d-Cellobiose, d-Lactose, d-l-Lactic Acid, d-malic acid, d-Mannitol, d-Melezitose, d-Melibiose, d-Trehalose, Glycyl-l-Glutamine, Glycyl-l-Proline, Inosine, Lactulose, l-Lactic Acid, l-malic acid, l-Methionine, l-Rhamnose, l-Valine, l-Valine plus l-Aspartic Acid, Maltotriose, myo-Inositol, Pyruvic Acid, Pyruvic Acid Methyl Ester, Stachyose, Succinic Acid Mono-Methyl Ester, Sucrose, Thymidine, Thymidine-5′-Monophosphate, Uridine and Uridine-5′-Monophosphate for growth. No growth occurred with 3-Methyl-d-Glucose, Acetic Acid, Adonitol, Alaninamide, Alanine arylamidase, Alkaline phosphatase, Amygdalin, Arbutin, Arginine arylamidase, Arginine dihydrolase, d-Arabitol, Dextrin, d-Fructose, dRaffinose, d-Galactose, d-Galacturonic acid, d-Glucosaminic Acid, d-Glucose-6-Phosphate, d-Glucuronic Acid, d-Lactic Acid Methyl Ester, d-l-α-Glycerol Phosphate, d-Mannose, d-saccharic acid, d-Sorbitol, Dulcitol, Formic Acid, Fumaric Acid, Glutamic acid decarboxylase, Glutamyl-glutamic acid arylamidase, Glycerol, Glycine arylamidase, Glycyl-l-Aspartic Acid, Glycyl-l-Methionine, Glyoxylic Acid, Histidine arylamidase, i-Erythritol, Itaconic Acid, l-Alanine, l-Alanyl-l-Glutamine, l-Alanyl-l-Histidine, l-Alanyl-l-Threonine, l-Asparagine, Leucine arylamidase, Leucyl-glycine arylamidase, l-Fucose, l-Glutamic Acid, l-Phenylalanine, l-Serine, l-Threonine, Mannose fermentation, m-Tartaric Acid, *N*-Acetyl-d-Galactosamine, *N*-Acetyl-d-glucosamine, *N*-Acetyl-β-d-Mannosamine, *N*-acetyl-β-glucosaminidase, Palatinose, Phenylalanine arylamidase, Proline arylamidase, Propionic Acid, Raffinose, Salicin, Serine arylamidase, Succinic Acid, Turanose, Tyrosine arylamidase and Urocanic Acid (Table S3).

Enzymatic assays for AGF001^T^ yielded positive reactions for esterase (C4), esterase lipase (C8), Acid phosphatase, α-Galactosidase, β-Galactosidase, α-glucosidase, β-glucuronidase and β-glucosidase. In contrast, negative results were obtained for: *N*-acetyl-β-glucosaminidase, Glutamic acid decarboxylase, α-Fucosidase, Nitrate reduction, Indole production, Urease, Alkaline phosphatase, α-chymotrypsin, for lipase (C14), naphthol-AS-BI-phosphohydrolase, *N*-acetyl-β-glucosaminidase, α-mannosidase, α-fucosidase, Arginine arylamidase, Proline arylamidase, Leucyl-glycine arylamidase, Phenylalanine arylamidase, Leucine arylamidase, Tyrosine arylamidase, Alanine arylamidase, Glycine arylamidase, Histidine arylamidase, Glutamyl-glutamic acid arylamidase, valine arylamidase, cystine arylamidase and Serine arylamidase.

Distinguishing phenotypic characteristics between strain AGF001^T^ and its closest relatives in the genus *Lacrimispora* (Table 3) revealed that, when compared to other members of the genus, strain AGF001^T^ was unique in its ability to utilize Fructose, Mannose, Salicin and Raffinose.

## Taxonomic conclusions

Based on polyphasic taxonomic criteria, OSA5^T^ and AGF001^T^ represent novel species within *Luteococcus* and *Lacrimispora*, respectively. It is proposed to name the novel strains OSA5^T^ and AGF001^T^ *Luteococcus struthious* sp. nov. and *Lacrimispora struthionigena* sp. nov.

## Description of *Luteococcus struthionis* sp. nov.

*Luteococcus struthionis* (stru.thi.o´nis. L. gen. n. *struthionis*, of an ostrich, *Struthio camelus*)

Cells are Gram-stain-positive, facultatively anaerobic, non-motile cocci (0.8–0.9 µm in diameter) and occur in pairs, tetrads, or clusters. Colonies are yellow, circular, and convex, on TSA after 48 h at 39 °C. Growth occurs at 4–50 °C (optimum 39 °C). The salinity range for growth is 0.0–9.0 % (w/v) NaCl (optimum 3.0 %), and the pH range for growth is pH 5.0–7.0 (optimum 6.0). Cells are catalase-positive. Growth occurred with Glucose, Fructose, Ribose, Xylose, Maltose, Sucrose, Melibiose, Gentiobiose, Stachyose, Pectin, Inosine, Mannitol, Sorbitol, Arabitol, Myo-inositol, Glycerol, Esculin, Gelatin, β-methyl-d-glucoside, d-salicin, *N*-acetyl neuraminic acid, Glucuronamide, Acetic acid, Propionic acid, Sodium butyrate, Mucic acid, Bromo-succinic acid, α-hydroxybutyric acid, α-ketobutyric acid, l-lactic acid, l-malic acid, l-alanine. Cells grew in the presence of lithium chloride, potassium tellurite, sodium bromate, Tween 40, nalidixic acid and aztreonam. Cells were positive for Alkaline phosphatase, Esterase (C4), Esterase lipase (C8), Leucine arylamidase, Trypsin, α-Chymotrypsin, Acid phosphatase, β-Glucuronidase, β-Galactosidase, α-Glucosidase, Arginine arylamidase, Pyrazinamidase, Pyrrolidonyl arylamidase and β-*N*-acetylglucosaminidase.

The type strain, OSA5^T^ (=CCM 9469^T^=CCUG 78312^T^=CECT 31229^T^=DSM 119679^T^), was isolated from ostrich faecal material collected from a private farm near Catoosa, Oklahoma (34.486589,–98.226482). The type strain’s DNA G+C content is 67.8 mol%. The GenBank accession numbers of the 16S rRNA gene and whole genome are PV166456.1 and GCA_048595755.1, respectively.

## Description of *Lacrimispora struthionigena* sp. nov.

*Lacrimispora struthionigena* (stru.thi.o.ni’ge.na. L. masc. n, *struthio*, an ostrich; N.L. masc. adj. suff. -*genus*, born from (from L. v. *gigno*, to produce, give birth to, beget); N.L. fem. adj. *struthionigena*, born of an ostrich)

Cells are Gram-stain-positive, obligately anaerobic, spore-forming, motile rods 3.5 x× 0.5 µm, and occur in singles. Colonies are white, circular and convex on TSAB after 48 h at 37 °C. Growth occurs at 25–45 °C (optimum 37 °C). The salinity range for growth is 0.5–2.0 % (w/v) NaCl (optimum 0.5%), and the pH range for growth is pH 6.0–8.0 (optimum pH 7.0). Growth occurred with 2′-Deoxy Adenosine, Lactose, d-l-Lactic Acid, d-Malic Acid, d-Mannitol, d-Melezitose, d-Melibiose, d-Trehalose, Glycyl-l-Glutamine, Glycyl-l-Proline, Inosine, Lactulose, l-Lactic Acid, l-Malic Acid, l-Methionine, l-Rhamnose, l-Valine, l-Valine plus l-Aspartic Acid, Maltotriose, myo-Inositol, Pyruvic Acid, Pyruvic Acid Methyl Ester, Stachyose, Succinic Acid Mono-Methyl Ester, Sucrose, Thymidine, Thymidine-5′-Monophosphate, Uridine and Uridine-5′-Monophosphate for growth. Cells were positive for esterase (C4), esterase lipase (C8), acid phosphatase, α-galactosidase, β-galactosidase, α-glucosidase, β-glucuronidase and β-glucosidase.

The type strain, and AGF001^T^ (=CCM 9471^T^=CCUG 78314^T^=CECT 31227^T^=DSM 119664^T^), was isolated from ostrich faecal material collected from a private farm near Catoosa, Oklahoma (34.486589,–98.226482). The type strain’s DNA G+C content is 41.6 mol%. The GenBank accession numbers of the 16S rRNA gene and whole genome are PV166457.2 and GCA_048627165.1, respectively.

## Supplementary material

10.1099/ijsem.0.007209 Supplementary Material 1.

## Abbreviations

ANI: average nucleotide identity
CCM: Czech Collection of Microorganisms
CCUG: Culture Collection University of Gothenburg
CECT: Spanish Type Culture Collection
dDDH: digital DNA–DNA hybridization
DSMZ: German Collection of Microorganisms and Cell Cultures
GH: glycoside hydrolase
GTDB: Genome Taxonomy Database
OGRI: overall genomic-based relatedness indices
RF: rumen fluid
TSA: tryptic soy agar
TSB: tryptic soy broth
