# Supplementary material for: Luteococcus struthionis sp. nov. and Lacrimispora struthionigena sp. nov. isolated from ostrich faeces
Source: Int J Syst Evol Microbiol. 2026 Jun 22;76(6):007209. doi: 10.1099/ijsem.0.007209 (PMC13286285; doi:10.1099/ijsem.0.007209)
Supplement: Supplementary Material 1. [file ijsem-76-07209-s001.pdf]

Supplementary Figure 1. Scanning electron microscope images of OSA5^T demonstrating cell shape (a) and arrangement (b). Scanning electron microscope images of AGF001^T demonstrating cell shape (c), arrangement (d), and endospore production (d).

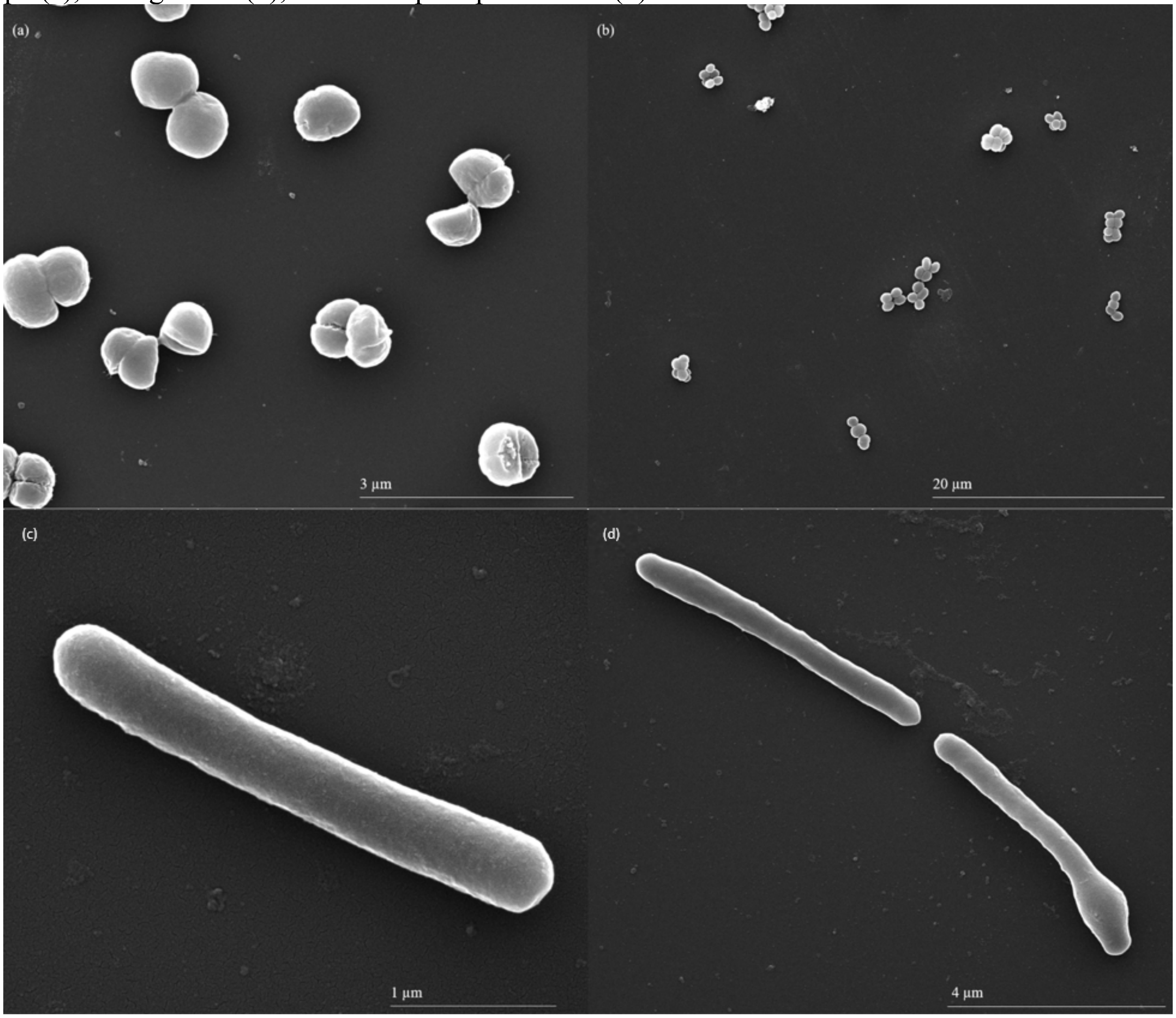

Supplementary Figure 2. CAZy content of strains OSA5^T and AGF001^T. (A) Pie charts showing the distribution of glycoside hydrolases, carbohydrate esterases, and polysaccharide lyases in the two strains. (B) Comparative analysis of the GH profile of strain OSA5^T compared to *Luetococcus* strains in NCBI RefSeq. (C) Comparative analysis of the GH profile of strain AGF001^T compared to *Lacrimispora* strains in NCBI RefSeq.

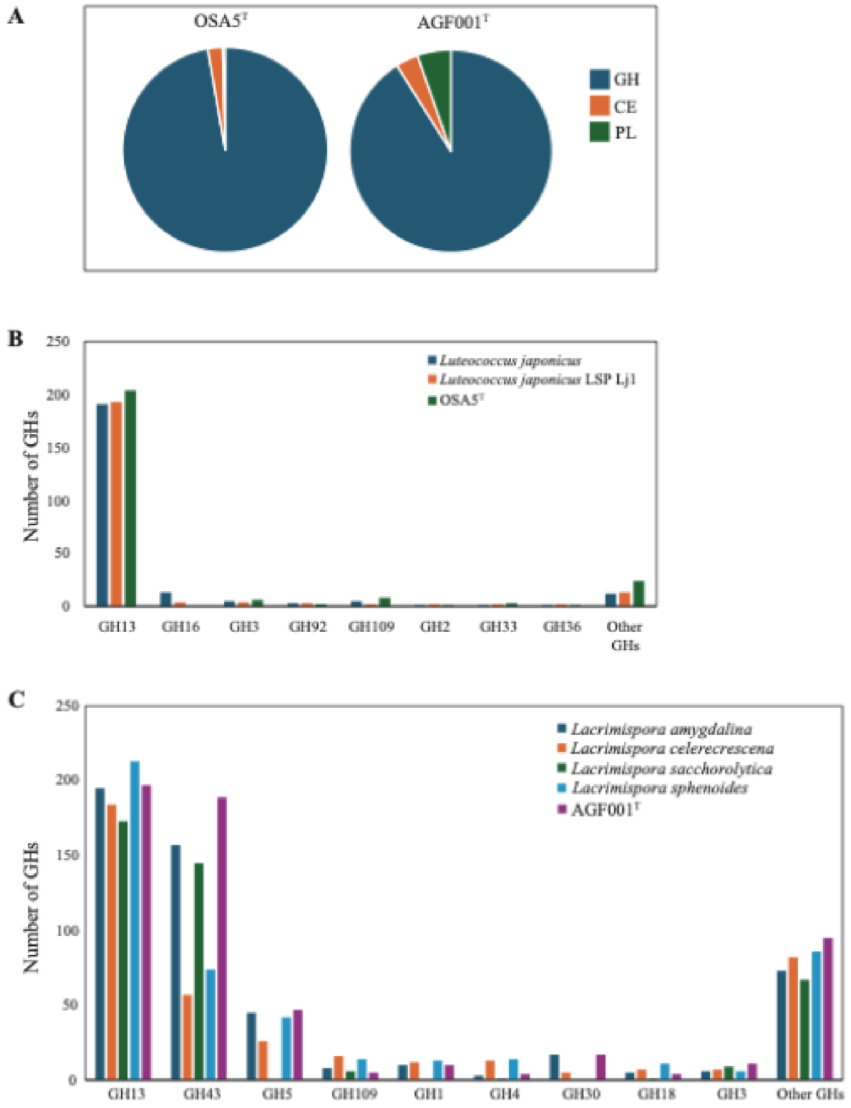

Supplementary Table 1. Genomic analysis of OSA5^{T} and AGF001^{T}.

Full pathway

Partial pathway

			GCF_048627165.1_Lacrimispora AGF001	GCF_048595755.1_Luteococcus OSA5
METABOLISM				
Carbohydrate metabolism				
	Central carbohydrate metabolism			
		M00001 Glycolysis (Embden-Meyerhof pathway), glucose => pyruvate (15) (complete 9/9)		
		M00002 Glycolysis, core module involving three-carbon compounds (8) (complete 5/5)		
		M00003 Gluconeogenesis, oxaloacetate => fructose-6P (12) (complete 7/7)		
		M00307 Pyruvate oxidation, pyruvate => acetyl-CoA (4) pyruvate-ferredoxin/flavodoxin oxidoreductase [EC:1.2.7.1 1.2.8.1]		
		M00009 Citrate cycle (TCA cycle, Krebs cycle) (20) (complete 8/8)		
		M00010 Citrate cycle, first carbon oxidation, oxaloacetate => 2-oxoglutarate (4) (complete 3/3)		
		M00011 Citrate cycle, second carbon oxidation, 2-oxoglutarate => oxaloacetate (16) (complete 5/5)		
		M00004 Pentose phosphate pathway (Pentose phosphate cycle) (9) (complete 6/6)		
		M00006 Pentose phosphate pathway, oxidative phase, glucose-6P => ribulose-5P (4) (complete 2/2)		
		M00007 Pentose phosphate pathway, non-oxidative phase, fructose-6P => ribose-5P (4) (complete 4/4)		
		M00005 PRPP biosynthesis, ribose-5P => PRPP (1) (complete 1/1)		
	Other carbohydrate metabolism			
		Propanoate degradation, Propanoate => succinate		
		M00631 D-Galacturonate degradation (bacteria), D-galacturonate => pyruvate + D-glyceraldehyde-3P (5) (complete 5/5)		

		M00061 D-Glucuronate degradation, D-glucuronate => pyruvate + D-glyceraldehyde-3P (6) (complete 5/5)		
		M00632 Galactose degradation, Leloir pathway, galactose => alpha-D-glucose-1P (4) (complete 4/4)		
		M00854 Glycogen biosynthesis, glucose-1P => glycogen/starch (4) (complete 2/2)		
		M00855 Glycogen degradation		
		M00565 Trehalose biosynthesis, D-glucose-1P => trehalose (6) (complete 6/6)		
		Rhamnose degradation, L-rhamnose => L-lactaldehyde + DHAP		
		Mannose degradation, D-mannose => Fructose-6-P		
		Fructose degradation, D-Fructose => Fructose-6-P		
		Xylose degradtion, xylose => D-ribulose 5P		
Energy metabolism				
	Fermentation and Substrate level phosphorylation			
		M00579 Phosphate acetyltransferase-acetate kinase pathway, acetyl-CoA => acetate (3) (complete 2/2)		
		acetyl-CoA synthetase [EC:6.2.1.1], Acetyl-CoA => Acetate		
		succinyl-CoA:acetate CoA-transferase [EC:2.8.3.18]		
		formate C-acetyltransferase [EC:2.3.1.54]		
		L-lactate dehydrogenase [EC:1.1.1.27]		
		Ethanol fermentation		
	Nitrogen metabolism			
		M00530 Dissimilatory nitrate reduction, nitrate => ammonia (5) (complete 2/2)		
		M00529 Denitrification		
	ATP synthesis			
		M00144 NADH:quinone oxidoreductase, prokaryotes (14) (complete 1/1)		
		M00148 Succinate dehydrogenase (ubiquinone)		
		M00151 Cytochrome bc1 complex respiratory unit (3) (complete 1/1)		
		M00153 Cytochrome bd ubiquinol oxidase		
		M00155 Cytochrome c oxidase, prokaryotes (4) (complete 1/1)		
		M00157 F-type ATPase, prokaryotes and chloroplasts (8) (complete 1/1)		
Lipid metabolism				
	Fatty acid metabolism			
		M00082 Fatty acid biosynthesis, initiation (7) (complete 2/2)		
		M00083 Fatty acid biosynthesis, elongation (4) (complete 1/1)		
	Lipid metabolism			

		M00089 Triacylglycerol biosynthesis		
		M00098 Acylglycerol degradation (2) (complete 2/2)		
Nucleotide metabolism				
	Purine metabolism			
		M00048 De novo purine biosynthesis, PRPP + glutamine => IMP (13) (complete 9/9)		
		M00049 Adenine ribonucleotide biosynthesis, IMP => ADP,ATP (4) (complete 4/4)		
		M00050 Guanine ribonucleotide biosynthesis, IMP => GDP,GTP (4) (complete 4/4)		
		M00053 Deoxyribonucleotide biosynthesis, ADP/GDP/CDP/UDP => dATP/dGTP/dCTP/dUTP (3) (complete 2/2)		
		M00958 Adenine ribonucleotide degradation, AMP => Urate (9) (complete 3/3)		
		M00959 Guanine ribonucleotide degradation, GMP => Urate (9) (complete 4/4)		
	Pyrimidine metabolism			
		M00051 De novo pyrimidine biosynthesis, glutamine (+ PRPP) => UMP (10) (complete 3/3)		
		M00052 Pyrimidine ribonucleotide biosynthesis, UMP => UDP/UTP,CDP/CTP (3) (complete 3/3)		
		M00938 Pyrimidine deoxyribonucleotide biosynthesis, UDP => dTTP (6) (complete 5/5)		
Amino acid metabolism				
	Alanine, aspartate and glutamate metabolism			
		aspartate ammonia-lyase [EC:4.3.1.1]		
	Glycne, Serine and threonine metabolism			
		M00020 Serine biosynthesis, glycerate-3P => serine (3) (complete 3/3)		
		M00018 Threonine biosynthesis, aspartate => homoserine => threonine (5) (complete 5/5)		
		M00621 Glycine cleavage system (3) (complete 3/3)		
		M00555 Betaine biosynthesis, choline => betaine (2) (complete 1/1)		
	Cysteine and methionine metabolism			
		M00021 Cysteine biosynthesis, serine => cysteine (2) (complete 2/2)		
		M00017 Methionine biosynthesis, aspartate => homoserine => methionine (8) (complete 7/7)		
	Branched-chain amino acid metabolism			
		M00019 Valine/isoleucine biosynthesis, pyruvate => valine / 2-oxobutanoate => isoleucine (5) (complete 4/4)		

		M00570 Isoleucine biosynthesis, threonine => 2-oxobutanoate => isoleucine (6) (complete 5/5)		
		M00432 Leucine biosynthesis, 2-oxoisovalerate => 2-oxoisocaproate (4) (complete 3/3)		
	Lysine metabolism			
		M00016 Lysine biosynthesis, succinyl-DAP pathway, aspartate => lysine (10) (complete 9/9)		
		M00526 Lysine biosynthesis, DAP dehydrogenase pathway, aspartate => lysine (6) (complete 6/6)		
		M00527 Lysine biosynthesis, DAP aminotransferase pathway, aspartate => lysine (7) (complete 7/7)		
	Arginine and proline metabolism			
		M00028 Ornithine biosynthesis, glutamate => ornithine (6) (complete 4/4)		
		M00844 Arginine biosynthesis, ornithine => arginine (3) (complete 3/3)		
		M00015 Proline biosynthesis, glutamate => proline (3) (complete 2/2)		
	Histidine metabolism			
		M00026 Histidine biosynthesis, PRPP => histidine (14) (complete 6/6)		
	Aromatic amino acid metabolism			
		M00023 Tryptophan biosynthesis, chorismate => tryptophan (8) (complete 3/3)		
		M00024 Phenylalanine biosynthesis, chorismate => phenylpyruvate => phenylalanine (5) (complete 2/2)		
		M00025 Tyrosine biosynthesis, chorismate => HPP => tyrosine (5) (complete 2/2)		
		M00878 Phenylacetate degradation		
		M00545 Trans-cinnamate degradation		
Metabolism of cofactors and vitamins				
	Cofactor and vitamin metabolism			
		M00899 Thiamine salvage pathway, HMP/HET => TMP (3) (complete 2/2)		
		M00125 Riboflavin biosynthesis, plants and bacteria, GTP => riboflavin/FMN/FAD (6) (complete 7/7)		
		M00120 Coenzyme A biosynthesis, pantothenate => CoA (6) (complete 3/3)		
		M00119 Pantothenate biosynthesis		
		M00881 Lipoic acid biosynthesis, plants and bacteria, octanoyl-ACP => dihydrolipoyl-E2/H (2) (complete 2/2)		
		M00126 Tetrahydrofolate biosynthesis, GTP => THF (8) (complete 5/5)		
		M00880 Molybdenum cofactor biosynthesis		
		M00988 PreQ1 biosynthesis, GTP => 7-Aminomethyl-7-deazaguanine (5) (complete 5/5)		
		M00140 C1-unit interconversion, prokaryotes (4) (complete 3/3)		

		M00846 Siroheme biosynthesis, glutamyl-tRNA => siroheme (6) (complete 6/6)		
		M00121 Heme biosynthesis, plants and bacteria, glutamate => heme (10) (complete 10/10)		
		M00926 Heme biosynthesis, bacteria, glutamyl-tRNA => coproporphyrin III => heme (9) (complete 9/9)		
		M00924 Cobalamin biosynthesis, anaerobic, uroporphyrinogen III => sirohydrochlorin => cobyrinate a,c-diamide (14) (complete 11/11)		
		M00925 Cobalamin biosynthesis, aerobic		
		M00116 Menaquinone biosynthesis		
Biosynthesis of terpenoids and polyketides				
	Terpenoid backbone biosynthesis			
		M00096 C5 isoprenoid biosynthesis, non-mevalonate pathway (8) (complete 8/8)		
		M00364 C10-C20 isoprenoid biosynthesis, bacteria (3) (complete 2/2)		
		M00365 C10-C20 isoprenoid biosynthesis, archaea (2) (complete 2/2)		
CELLULAR PROCESSES				
	ko02035 Bacterial motility proteins			
		Bacterial chemotaxis		
		Flagellar assembly		
	ko02022 Two-component system			
	OmpR family			
		PhoR-PhoB (phosphate starvation response)		
		SenX3-RegX3 (phosphate starvation response)		
		KdpD-KdpE (potassium transport)		
		MprB-MprA (maintenance of persistent infection)		
		MtrB-MtrA (osmotic stress response)		
	NarL family			
		DesK-DesR (membrane lipid fluidity regulation)		
	LytTR family			
		ko02035 Bacterial motility proteins		
		AgrC-AgrA (exoprotein synthesis)		
	Other families			
		YesM-YesN		
		PdtaS-PdtaR		

	CheA family			
		CheA-CheYBV (chemotaxis)		

Supplementary Table 2. Metabolic traits for OSA5^T using GENIII Biolog microplate. Positive results are in bold.

Phenotypic Test Results			
Dextrin	-	Glycyl-L-Proline	-
**D-Maltose**	+	**L-Alanine**	+
**D-Trehalose**	+	L-Arginine	-
D-Cellobiose	-	L-Aspartic Acid	-
**Gentiobiose**	+	L-Glutamic Acid	-
**Sucrose**	+	L-Histidine	-
**D-Turanose**	+	L-Pyroglutamic Acid	-
**Stachyose**	+	L-Serine	-
**pH 6**	+	Lincomycin	-
**pH 5**	+	Guanidine HCl	-
**D-Raffinose**	+	Niaproof 4	-
α-D-Lactose	-	**Pectin**	+
**D-Melibiose**	+	D-Galacturonic Acid	-
**β-Methyl-D-Glucoside**	+	L-Galactonic Acid Lactone	-
**D-Salicin**	+	D-Gluconic Acid	-
N-Acetyl-D-Glucosamine	-	D-Glucuronic Acid	-
N-Acetyl-β-D-Mannosamine	-	**Glucuronamide**	+
N-Acetyl-D-Galactosamine	-	**Mucic Acid**	+
**N-Acetyl Neuraminic Acid**	+	Quinic Acid	-
**1% NaCl**	+	D-Saccharic Acid	-
**4% NaCl**	+	Vancomycin	-
**8% NaCl**	+	Tetrazolium Violet	-
**α-D-Glucose**	+	Tetrazolium Blue	-
**D-Mannose**	+	p-Hydroxy-Phenylacetic Acid	-
**D-Fructose**	+	Methyl Pyruvate	-
D-Galactose	-	D-Lactic Acid Methyl Ester	-
3-Methyl Glucose	-	L-Lactic Acid	+
D-Fucose	-	Citric Acid	-
L-Fucose	-	α-Keto-Glutaric Acid	-
L-Rhamnose	-	D-Malic Acid	-
**Inosine**	+	**L-Malic Acid**	+
**1% Sodium Lactate**	+	**Bromo-Succinic Acid**	+
Fusidic Acid	-	**Nalidixic Acid**	+
**D-Serine**	+	**Lithium Chloride**	+
**D-Sorbitol**	+	**Potassium Tellurite**	+
**D-Mannitol**	+	**Tween 40**	+
**D-Arabitol**	+	γ-Amino-Butyric Acid	-
**myo-Inositol**	+	**α-Hydroxy-Butyric Acid**	+
**Glycerol**	+	β-Hydroxy-D,L-Butyric Acid	-

D-Glucose-6-PO4	-	**α-Keto-Butyric Acid**	+
D-Fructose-6-PO4	-	Acetoacetic Acid	-
D-Aspartic Acid	-	**Propionic Acid**	+
D-Serine	-	**Acetic Acid**	+
Troleandomycin	-	Formic Acid	-
Rifamycin SV	-	**Aztreonam**	+
Minocycline	-	**Sodium Butyrate**	+
Gelatin	-	**Sodium Bromate**	+

Supplementary Table 3. Metabolic traits for AGF001^{T} using the AN Biolog microplate. Positive results are in bold.

Phenotypic Test Results			
N-Acetyl-D-Galactosamine	-	Turanose	-
N-Acetyl-D-Glucosamine	-	Acetic Acid	-
N-Acetyl-β-D-Mannosamine	-	Formic Acid	-
Adonitol	-	Fumaric Acid	-
Amygdalin	-	Glyoxylic Acid	-
D-Arabitol	-	α-Hydroxybutyric Acid	-
Arbutin	-	β-Hydroxybutyric Acid	-
**D-Cellobiose**	+	Itaconic Acid	-
α-Cyclodextrin	-	α-Ketobutyric Acid	-
β-Cyclodextrin	-	**α-Ketovaleric Acid**	+
Dextrin	-	**D-L-Lactic Acid**	+
Dulcitol	-	**L-Lactic Acid**	+
i-Erythritol	-	D-Lactic Acid Methyl Ester	-
D-Fructose	-	**D-Malic Acid**	+
L-Fucose	-	**L-Malic Acid**	+
D-Galactose	-	Propionic Acid	-
D-Galacturonic Acid	-	**Pyruvic Acid**	+
D-Glucosaminic Acid	-	**Pyruvic Acid Methyl Ester**	+
D-Glucuronic Acid	-	D-Saccharic Acid	-
D-Glucosaminic Acid	-	Succinic Acid	-
α-D-Glucose-1-Phosphate	-	Succinic Acid	-
D-Glucose-6-Phosphate	-	**Succinic Acid Mono-Methyl Ester**	+
3-Methyl-D-Glucose	-	m-Tartaric Acid	-
Glycerol	-	Urocanic Acid	-
D-L-α-Glycerol Phosphate	-	Alaninamide	-
**myo-Inositol**	+	L-Alanine	-
**D-Lactose**	+	L-Alanyl-L-Glutamine	-
**Lactulose**	+	L-Alanyl-L-Histidine	-
**Maltotriose**	+	L-Alanyl-L-Threonine	-
**Maltotriose**	+	L-Asparagine	-
**D-Mannitol**	+	L-Glutamic Acid	-
D-Mannose	-	L-Glutamic Acid	-
**D-Melezitose**	+	Glycyl-L-Aspartic Acid	-
**D-Melibiose**	+	**Glycyl-L-Glutamine**	+
**3-Methyl-D-Glucose**	+	Glycyl-L-Methionine	-
α-Methyl-D-Galactoside	-	**Glycyl-L-Proline**	+

β-Methyl-D-Galactoside	-	**L-Methionine**	+
α-Methyl-D-Glucoside	-	L-Phenylalanine	-
β-Methyl-D-Glucoside	-	L-Serine	-
Palatinose	-	L-Threonine	-
**D-Raffinose**	+	**L-Valine**	+
**L-Rhamnose**	+	**L-Valine plus L-Aspartic Acid**	+
Salicin	-	**2'-Deoxy Adenosine**	+
D-Sorbitol	-	**Inosine**	+
**Stachyose**	+	**Thymidine**	+
**Sucrose**	+	**Uridine**	+
**D-Trehalose**	+	**Thymidine-5'-Monophosphate**	+
		**Uridine-5'-Monophosphate**	+
